# Alteration in the Gut Microbiota of Chickens Resistant to *Eimeria tenella* Infection

**DOI:** 10.3390/microorganisms12112218

**Published:** 2024-10-31

**Authors:** Yu Qiao, Qian Feng, Qingjie Wang, Qiping Zhao, Shunhai Zhu, Fanghe Zhao, Zhongchuang Wang, Ruiting Zhang, Jinwen Wang, Yu Yu, Hongyu Han, Hui Dong

**Affiliations:** 1 Shanghai Veterinary Research Institute, Chinese Academy of Agricultural Sciences, Key Laboratory of Animal Parasitology of Ministry of Agriculture, Minhang, Shanghai 200241, China; qiaoyu540447420@163.com (Y.Q.); 15248214560@163.com (Q.F.); wangwqj21006@163.com (Q.W.); zqp@shvri.ac.cn (Q.Z.); zhushunhai@shvri.ac.cn (S.Z.); xzfh824@163.com (F.Z.); zxclkjhf1999601@163.com (Z.W.); 2218302044@st.gxu.edu.cn (R.Z.); 82101221311@caas.cn (J.W.); yy21058@163.com (Y.Y.); hhysh@shvri.ac.cn (H.H.); 2 College of Animal Science and Technology, Guangxi University, Nanning 530004, China

**Keywords:** *Eimeria tenella*, cecal microbiota, fecal microbiota transplantation

## Abstract

Avian coccidiosis, caused by several species of *Eimeria*, is a widespread and economically important poultry disease that inflicts severe losses in the poultry industry. Understanding the interplay between *Eimeria* and gut microbiota is critical for controlling coccidiosis and developing innovative treatments to ensure good poultry health. In the present study, chickens were immunized six times with a low dose of *Eimeria tenella*, resulting in complete immunity against *Eimeria* infection. The results of fecal microbiota transplantation showed that the gut microbiota of immunized chickens induced a certain degree of resistance to coccidial infection. To investigate the types of intestinal microbiota involved in the development of resistance to *Eimeria*, the intestinal contents and fecal samples from both immunized and unimmunized groups were collected for 16S rRNA gene sequencing. The results showed that, at the genus level, the abundance of the *Eubacterium coprostanoligenes* group, *Erysipelatoclostridium*, *Shuttleworthia*, and *Colidextribacter* was significantly increased in the intestinal content of immunized chickens, whereas the abundance of *Eisenbergiella* was significantly decreased. In fecal samples, the abundance of Clostridiaceae and Muribaculaceae significantly increased, whereas that of Bacillales significantly decreased. These findings will help to elucidate the interactions between *E. tenella* and the gut microbiota of chickens, providing a basis for isolating *E. tenella*-resistant strains from the gut microbiome and developing new vaccines against coccidiosis.

## 1. Introduction

Microbiota refers to all microorganisms, including bacteria, yeasts, filamentous fungi, and viruses, that inhabit different body parts of all animals and play a crucial role in the development and functioning of the immune system [1]. Each animal species has coevolved in nature with its microbial symbionts, and this relationship offers survival advantages to both the host and its associated microbial communities. The gastrointestinal tract (GIT) of the chicken comprises a complex and diverse microbiota that shows a beneficial symbiotic interaction with the host, including promoting the maturation of the gut immune system and thereby protecting the host from intestinal infections caused by pathogenic or opportunistic enteric microorganisms [2,3]. Maintaining a healthy intestinal environment is essential for the efficient performance of chickens [4]. However, chickens constantly face the risk of poor health due to infections caused by a series of poultry pathogens such as parasitic protozoa [5].

Coccidiosis, caused by protozoa of the genus *Eimeria*, is among the most significant parasitic diseases in the poultry industry; it poses a critical global challenge and results in substantial economic losses [6]. Thus far, seven *Eimeria* species have been recognized in chickens; moreover, although these species exhibit significant differences in their parasitic location and virulence, they have a similar life cycle [7]. *Eimeria* infection not only disrupts the structure of the intestinal tissues and lining but also destabilizes the microbial community in the GIT; these conditions promote colonization and proliferation of other opportunistic pathogens such as *Clostridium perfringens*, leading to susceptibility of the infected chickens to secondary diseases and thereby increasing mortality of chickens [8]. Hence, it is critical to understand the interplay between *Eimeria* and gut microbiota to control and prevent enteric diseases and develop innovative treatment approaches for ensuring good poultry health [9].

*Eimeria tenella* is one of the most prevalent and harmful species of *Eimeria* in poultry farming; it parasitizes the cecum of chickens, which harbors gut microbiota, including Firmicutes, *Bacteroidetes*, and Proteobacteria [10]. Zhou et al. reported that *E. tenella* infection substantially altered the composition and diversity of the cecal microbiota, particularly by reducing the abundance of Proteobacteria and Firmicutes [11]. *E. tenella* infection also caused a shift in the gut microbiota and decreased cecal microbial diversity in chickens [12]. Chen et al. observed that the abundance of *Lactobacillus*, *Faecalibacterium*, *Ruminococcaceae* UCG-013, *Romboutsia*, and *Shuttleworthia* decreased during *E. tenella* infection [9]. Additionally, some researchers observed changes in the gut microbiota after different chicken strains were infected with *E. tenella*. The abundance of Firmicutes and *Bacteroidetes* decreased, while that of Proteobacteria increased significantly in the different chicken lines infected with *E. tenella* [10]. These studies suggest that *E. tenella* infection alters the composition of the cecal gut microbiota. However, these studies used relatively high infection doses, resulting in severe pathological changes and significant damage to the intestinal mucosa, which reflected changes in the gut microbiota under a diseased state. To date, no studies have investigated changes in the gut microbiota of chickens with strong resistance to *E. tenella*.

Hence, the present study was conducted to determine changes in the intestinal bacterial microbiota of chickens exhibiting strong resistance to *E. tenella* infection. The results of this study could help us understand the interactions between *Eimeria* infection and the gut microbiota.

## 2. Materials and Methods

### 2.1. Parasite Strain, Animals, and Ethics Statement

The Shanghai strain of *E. tenella* (Resource Number CAAS21111601) was initially isolated in the 1980s from a sample collected on a farm in Shanghai, China, and has since been maintained in our laboratory [13]. Coccidia-free, 1-day-old healthy three-yellow chickens were purchased from the Shanghai Minyou Poultry Farming Professional Cooperative. Chickens were raised in a temperature-controlled, coccidia-free environment until they reached 7 days of age. After this period, the chickens were transferred to experimental growth cages. Prior to the trial’s conclusion, they had unrestricted access to fresh, clean water and nonmedicated commercial feed.

All animal procedures were conducted under the approval of the Animal Ethics Committee of the Shanghai Veterinary Institute, Chinese Academy of Agricultural Sciences. The experiments adhered to animal ethics guidelines and approved protocols (Permit Number: SHVRI-SZ-20230323-4).

### 2.2. Immunization with E. tenella and Evaluation of Immunization Efficacy

Forty 8-day-old chickens were divided into two groups, an immunized group and a unimmunized group, with 20 chickens in each group. The immunized group was immunized with sporulated oocysts of the *E. tenella* strain at 8, 15, 22, 29, 36, and 43 days of age; thus, 6 immunizations were performed at the dose of 5000 oocysts per chicken.

On the 7th day after the sixth immunization (i.e., at 49 days of age), five chickens each from the immunized and unimmunized groups were randomly selected and challenged with sporulated oocysts of *E. tenella* at the dose of 3 × 10^4^ sporulated oocysts per chicken. Five chickens from the unimmunized group were used as the unimmunized and unchallenged group.

Fecal samples were collected daily from days 6 to 7 post-challenge. The number of fecal oocysts was determined using a McMaster chamber according to the established methods. On the 8th day after the challenge, intestinal lesions in the chickens were scored according to the method of Johnson and Reid [14]. The cecum was collected, and the integrity of the intestinal villi was observed under a microscope following fixation of the cecal tissue with 10% formalin, dehydration, paraffin embedding, sectioning, hematoxylin and eosin (H&E) staining, and other necessary procedures.

### 2.3. Evaluation of the Anticoccidial Effect of the Intestinal Flora of Immunized Chickens

Fecal microbiota transplantation (FMT) was performed to evaluate the anticoccidial effect of the chicken intestinal flora after 6 immunization doses. Five hundred grams of fresh feces of immunized chickens was collected and suspended in sterile 0.9% saline solution; the mixture was then stirred and filtered through a sterile gauze. The filtrate was centrifuged at 3000 rpm for 5 min, and the sediment was subsequently resuspended in sterile 0.9% saline and washed twice. Finally, an appropriate amount of sterile saline was added to obtain a final concentration of 10 g of fecal bacteria/mL. Serial dilutions were performed to obtain solutions containing 5 g and 1 g of fecal bacteria/mL. The entire procedure was performed on ice [15].

Next, 80 one-day-old chicks were divided into 8 groups: 6 immunized challenge groups, 1 unimmunized challenge group, and 1 unimmunized unchallenged group, with 10 chickens in each group. When chickens were 1 day old, the immunized challenge groups were inoculated with 1, 5, or 10 g/chicken through the cloaca or gavage, and the unimmunized challenge groups were inoculated with equal amounts of PBS. After 7 days, the second immunization dose identical to the first dose was administered. At 7 days after the second immunization, each chicken in the 6 immunized challenge groups and the unimmunized challenge group was infected with 3 × 10^4^ fresh sporulated oocysts of *E. tenella*. The number of oocysts in feces was counted at 6–8 days after the challenge. The reduction in oocyst number was calculated using the following formula: (number of oocysts from the unimmunized challenge group − number of oocysts from the immunized challenge group)/number of oocysts from the unimmunized challenge group × 100% [16]. On the 8th day after the challenge, the ceca of each group were collected separately, and the intestinal lesions were scored according to the method of Johnson and Reid [14].

### 2.4. Microbiome Sequencing and Bioinformatics Analysis

On the 14th day after six immunization doses, the microbiome of cecal contents and fresh feces from the remaining chickens was analyzed. Eight chickens were randomly selected from the immunized and unimmunized groups for collection of fresh feces and cecal contents. The fresh feces and cecal contents of the immunized group were designated as IMF01–IMF08 and IMC01–IMC08, respectively, and the fresh feces and cecal contents of the unimmunized group were designated as CF01–CF08 and CC01–CC08, respectively.

To assess the fecal microbiota profile through 16S rRNA gene sequencing, total genomic DNA was extracted from the samples using the cetyltrimethylammonium bromide-sodium dodecyl sulfate method, as described previously [17]. DNA concentration and purity were monitored on 1% agarose gels. The V3–V4 hypervariable region of the 16S rRNA gene was amplified with a forward primer (338F: 5′-ACTCCTRCGGGAGGCAGCAG-3′) and a reverse primer (806R: 5′-GGACTACCVGGGTATCTAAT-3′) [18]. PCR products were run in an electrophoresis chamber on a 2% agarose gel and purified using the AxyPrep DNA Gel Extraction Kit (Axygen Biosciences, Union City, CA, USA) in accordance with the manufacturer’s instructions and quantified using QuantiFluor™-ST (Promega, Madison, WI, USA). Purified amplicons were used for library preparation and pyrosequencing. Sequencing libraries were generated using the NEBNext^®^ Ultra™ DNA Library Prep Kit (New England Biolabs, Ipswich, MA, USA) in accordance with the manufacturer’s instructions. Library quality was assessed and sequenced on an Illumina MiSeq platform (PE300).

Taxonomic classification of the operational taxonomic units (OTUs) was conducted by performing a BLAST search against the Greengenes database [19,20]. Subsequently, alpha and beta diversity indices were calculated using QIIME2 software(version 2022.5). Alpha diversity was evaluated using indices such as Chao1, Shannon, Simpson, and Observed Species, while beta diversity was assessed through Bray–Curtis principal coordinate analysis (PCoA) and statistical analysis with PERMANOVA. Differentially abundant taxa across the experimental groups were determined through linear discriminant analysis effect size (LEfSe) with default parameters [21].

### 2.5. Statistical Analysis

All results are presented as mean ± standard deviation. All data were statistically analyzed by Student’s *t*-test. Differences with *p* < 0.05 were considered statistically significant.

## 3. Results

### 3.1. Results of Evaluation of Immunization Efficacy

Immunization efficacy following six immunization doses with *E. tenella* was assessed by challenging the chickens with 3 × 10^4^ *E. tenella* sporulated oocysts. The results showed that the unimmunized challenge group produced many oocysts at 6–7 days post-challenge and exhibited moderate intestinal lesions; in contrast, the immunized challenge group exhibited no oocyst discharge and had an intestinal lesion score of zero (Table 1).

**Figure 1:**
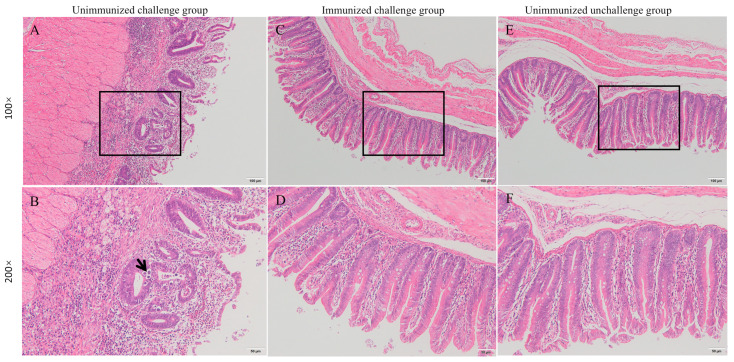
Histopathological images of the cecum.Cecal sections of the unimmunized challenge group at 100× magnification (**A**) and 200× magnification (**B**). Cecal sections of the immunized challenge group at 100× magnification (**C**) and 200× magnification (**D**). Cecal sections of the unimmunized unchallenged group at 100× magnification (**E**) and 200× magnification (**F**). Oocysts are indicated by arrows.

Histopathological analysis revealed that chickens in the immunized challenge group had neatly arranged, intact tissue structures. The epithelial cells of the intestinal glands were closely adhered to the basement membrane, and their structural integrity was maintained. No edema or ulceration was observed. The intestinal glands appeared abundant and closely arranged. In contrast, the unimmunized challenge group showed pronounced coccidiosis characterized by swollen and blunt intestinal villi, with developing oocysts visibly embedded within the tissues (Figure 1).

### 3.2. Results of the Anticoccidial Effect of the Intestinal Flora of Immunized Chickens

Following challenge with oocysts, fecal samples were collected on days 6, 7, and 8 to determine the oocyst yield for each group (Table 2). The results revealed a significant reduction in oocyst production in the immunized challenge groups (1, 5, and 10 g), with the decrease ranging from 6.4% to 44.2%. The 1 g through gavage group and the 5 g through cloaca group exhibited a higher reduction in oocyst production (44.2% and 31.3%, respectively).

On the 8th day post-challenge, the ceca were examined to record intestinal lesions in each group (Table 2). The results indicated that the number of intestinal lesions in the 5 g through cloaca, 10 g through cloaca, and 1 g through gavage groups (+2.4, +2.1, and +2.3, respectively) was significantly lower than that in the unimmunized challenge group (+2.9).

### 3.3. Results of Intestinal Microbiota Analysis Following Immunization

#### 3.3.1. *E. tenella* Changed Gut Microbiota Diversity

**Figure 2:**
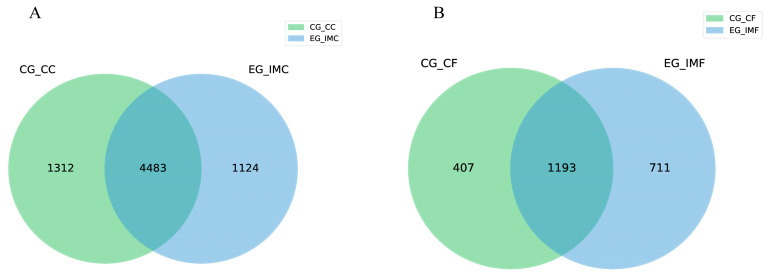
(**A**) Venn diagram showing the number of OTUs in the cecal contents of the unimmunized group (CG-CC) and the immunized group (EG-IMC). (**B**) Venn diagram showing the number of OTUs in the feces of the unimmunized group (CG-CF) and the immunized group (EG-IMF).

To evaluate the effect of *E. tenella* immunization on the diversity and richness of the intestinal flora, OTUs in cecal contents and feces of the immunized and unimmunized groups were identified. The average number of OTUs (5607) observed in the intestinal content of the immunized group after *E. tenella* immunization was slightly lower than that in the unimmunized group (5795); however, the difference was not significant. The mean OTU number in feces (1904) was higher in the immunized group than in the unimmunized group (1600) (Figure 2).

**Figure 3:**
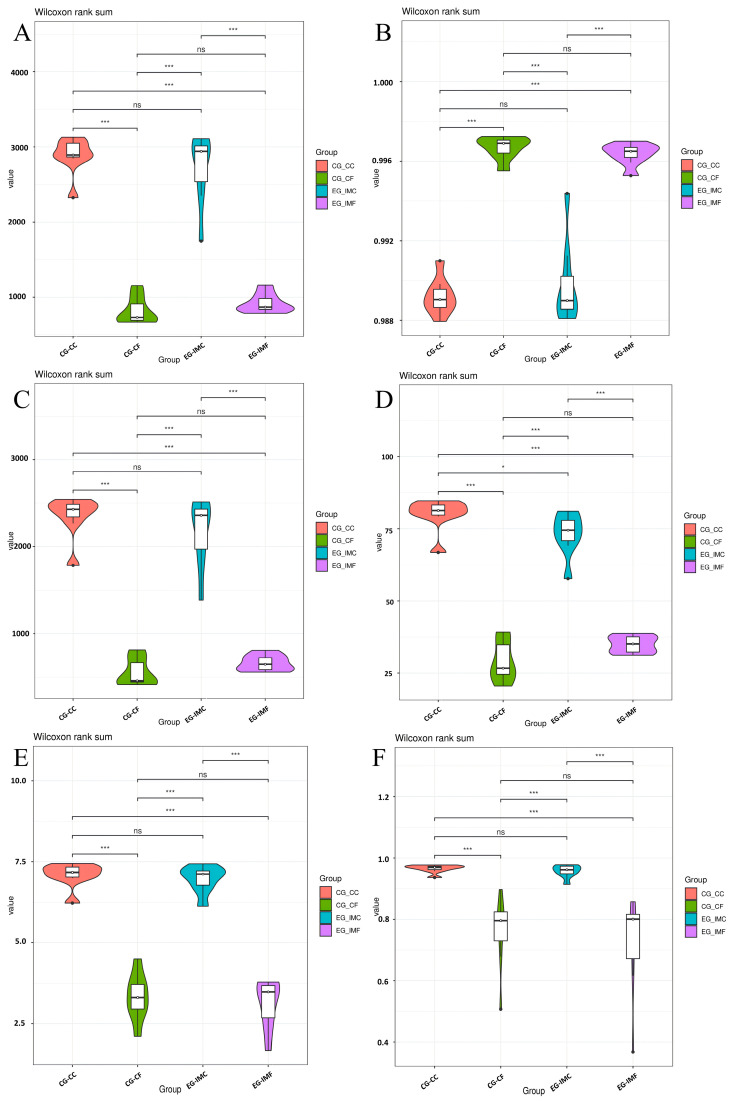
Alpha diversity analysis. (**A**) Chao1 index, (**B**) Good’s coverage, (**C**) Observed Species, (**D**) PD_whole_tree index, (**E**) Shannon index, (**F**) Simpson index. * means *p* < 0.05, *** means *p* < 0.001, ns means no statistical differences.

Alpha diversity analysis was performed on the sequence data by using the number of OTUs, Chao1 index, Good’s coverage, Shannon index, and other metrics. The results are presented in Figure 3 and Table 3. The immunized and unimmunized groups showed no differences in Chao1, Good’s coverage, Shannon, Observed Species, and Simpson indices of the microbiota from the cecal contents and feces. However, the PD_whole_tree index of the cecal content was significantly lower in the immunized group than in the unimmunized group (*p* < 0.05). These results suggest that the diversity of the chicken intestinal flora after immunization was affected by *E. tenella* invasion; however, the difference was not significant (Figure 3).

**Figure 4:**
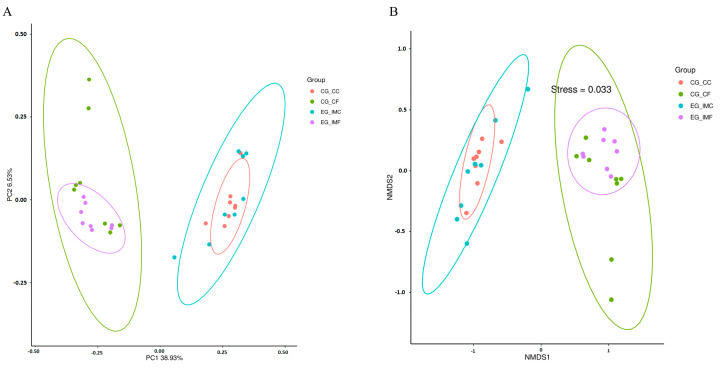
Beta diversity analysis. (**A**) PCoA analysis and (**B**) NMDS analysis.

Beta diversity analysis was performed on the sequence data using the PCoA and nonmetric multidimensional scaling (NMDS). The results showed that the unweighted UniFrac distances from the PCoA and NMDS analyses indicated significant nonclustering of the microbial species from the intestinal contents or feces between the immunized and unimmunized groups, thus indicating differences in their microbial community composition (Figure 4). These findings further support the hypothesis that the chicken intestinal flora diversity changed after immunization.

#### 3.3.2. *E. tenella* Altered Bacterial Taxa in the Gut

**Figure 5:**
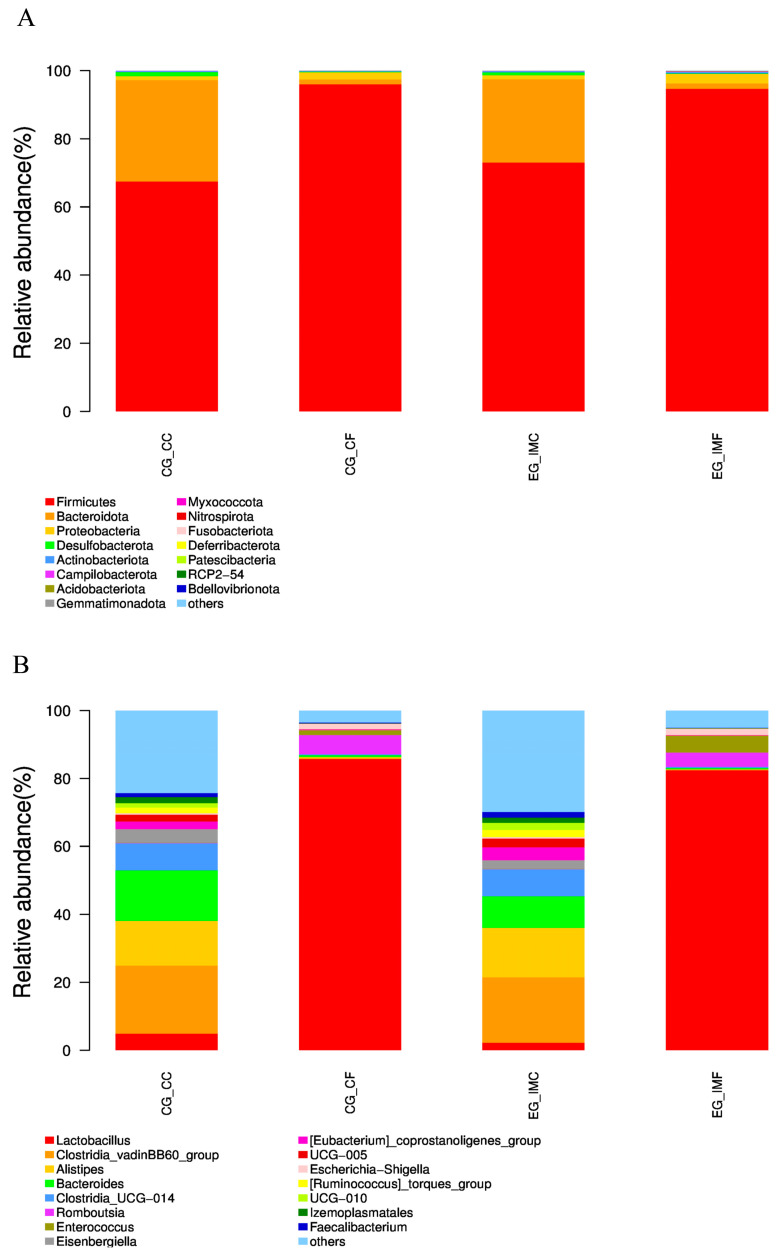
The bacterial flora composition in the intestinal content (CG-CC and EG-IMC) and feces (CG-CF and EG-IMF) before and after *E. tenella* immunization.(**A**) Taxa assignments of 4 groups at the phylum level. (**B**) Taxa assignments of 4 groups at the genus level.

At the phylum level, the overall intestinal flora composition of each group is shown in Figure 5A. The bacterial community structure of the cecal flora differed significantly from that of the fecal flora. Fecal bacteria in the unimmunized group (CG-CF) mainly included Firmicutes (95.96%), Proteobacteria (2.12%), and Bacteroidota (1.43%). In the feces of the immunized group (EG-IMF), the abundance of Firmicutes (94.65%) decreased, whereas that of Proteobacteria (2.84%) and Bacteroidota (1.57%) increased. The cecal content of the unimmunized group (CG-CC) contained Firmicutes (67.44%), Bacteroidota (29.77%), Proteobacteria (1.17%), and Desulfobacterota (1.14%). In the immunized group, the abundance of Firmicutes (73.01%) and Proteobacteria (1.20%) in the cecal content (EG-IMC) increased, whereas that of Bacteroidota (24.44%) and Desulfobacterota (0.79%) decreased.

At the genus level, the overall intestinal flora composition of each group is shown in Figure 5B. The microbial community structure of the cecal content and fecal flora significantly differed. In the unimmunized group, CG-CF mainly included *Lactobacillus* (85.71%), *Romboutsia* (5.67%), and *Enterococcus* (1.42%). In the feces of the immunized group (EG-IMF), the abundance of *Enterococcus* (4.97%) increased, whereas that of *Lactobacillus* (82.38%) and *Romboutsia* (4.22%) decreased. The cecal content flora (CG-CC) in the unimmunized group mainly included *Clostridia_vadinBB60*_group (20.05%), *Alistipes* (13.20%), and *Bacteroides* (14.77%). In the immunized group, the abundance of *Alistipes* increased to 14.57%, whereas that of the *Clostridia_vadinBB60*_group (19.29%) and *Bacteroides* (9.31%) decreased.

#### 3.3.3. Differential Flora Screening Results

**Figure 6:**
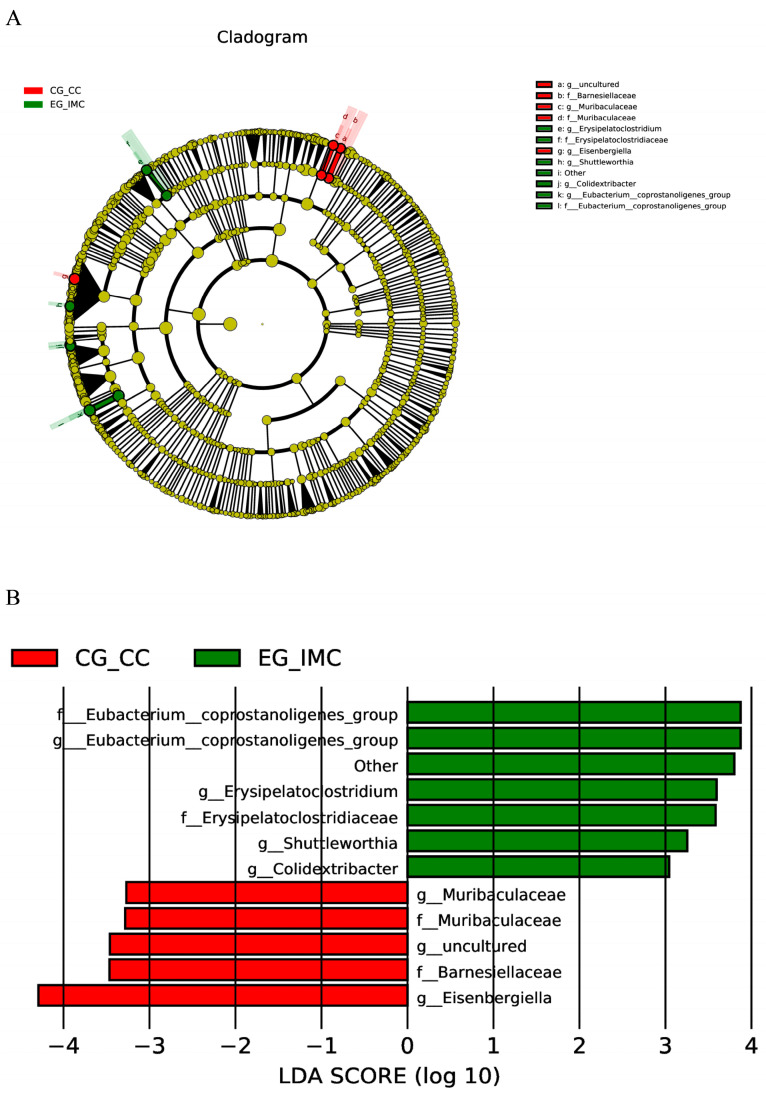
LEfSe analysis of the microbial flora in the intestinal contents of the unimmunized groups (CG-CC) and the immunized groups (EG-IMC). An LDA effect size of >3 was used as a threshold for the LEfSe analysis. (**A**) LEfSe analysis branch diagram (**B**) LDA scores obtained from LEfSe analysis.

**Figure 7:**
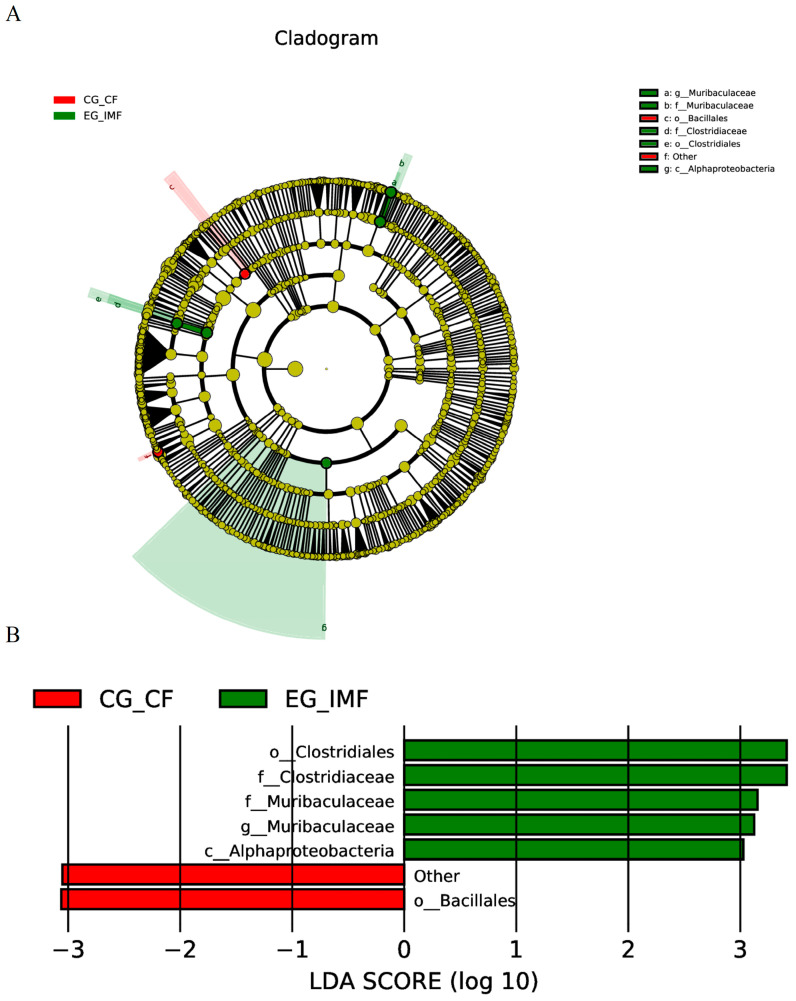
LEfSe analysis of the bacterial flora in the feces of the unimmunized groups (CG-CF) and the immunized groups (EG-IMF). An LDA effect size of >3 was used as a threshold for the LEfSe analysis. (**A**) LEfSe analysis branch diagram. (**B**) LDA scores obtained from LEfSe analysis.

To completely understand the effects of *E. tenella* immunization on the bacterial flora in the cecal content and feces, we performed LEfSe analysis of the microbial flora detected in the cecal content and feces of the immunized and unimmunized groups. Figure 6 and Figure 7 show the classification cladograms and LDA scores obtained by LEfSe analysis.

Eleven bacterial groups were identified with differences between the cecal content of the immunized and unimmunized groups. Among these groups, 7 genera had LDA scores > 3 (Figure 6A,B). In the immunized group, the intestinal content showed a significant increase in the abundance of genera such as *Eubacterium coprostanoligenes* group, *Erysipelatoclostridium*, *Shuttleworthia*, and *Colidextribacter*, while the abundance of Muribaculaceae and *Eisenbergiella* significantly decreased. Six bacterial groups showed differences between the abundance in feces of the immunized and unimmunized groups (Figure 7A,B). Among these groups, the abundance of Clostridiaceae, Clostridiales, Muribaculaceae, and Alphaproteobacteria increased significantly, while that of Bacillales decreased significantly.

## 4. Discussion

Coccidiosis causes substantial economic losses to the farming industry and poses a major risk to global poultry production. Understanding the intestinal microbial composition and structure in chickens throughout the life cycle of coccidial infection may help identify correlations between alterations in the gut microbiota and protozoan invasion over time; this may further enable discovery of potential biomarkers and facilitate the development of novel practical treatment methods [22]. Therefore, the present study examined changes in the intestinal flora of chickens after they acquired complete resistance to *E. tenella* infection. The findings of this study could enable us to better comprehend the interactions between *Eimeria* infection and gut microbiota and provide a basis for isolating *E. tenella*-resistant strains of the gut microbiome and developing new vaccines.

First, a relatively low-infected dose of *E. tenella*, with a small impact on chicken intestinal morphology, was used to immunize chickens against *Eimeria* infection. The animal challenge experiment with a high dose of *E. tenella* showed that no oocysts were detected in the feces and no intestinal lesions were observed in the cecum of the immunized chickens; moreover, the intestinal tissues and mucosa remained relatively intact, indicating that the chickens had gained complete immunity against *E. tenella* after six sessions of immunization. These results were consistent with those of previous studies [23].

FMT is a well-known method for altering the composition of gut microorganisms in the context of diseases [24]. FMT involves the introduction (transplantation) of the intestinal microbiota from the feces of a healthy donor into the gastrointestinal tract of a patient. This method is most commonly used for treating gastrointestinal diseases caused by pathogenic or conditionally pathogenic microorganisms [25,26]. Recent studies have indicated that the evolutionary capacity of probiotic microorganisms may also serve as a novel therapeutic strategy for combating various diseases [27]. In the present study, FMT was used to feed the microbiota from fresh feces of immunized chickens to one-day-old chicks to investigate whether this acquired resistance to *Eimeria* was associated with changes in the intestinal microbiota. The results revealed that oocyst production and cecal lesions in the immunized chickens were reduced to varying degrees. These findings suggest that the gut microbiota of chickens with complete immunity against *E. tenella* could induce the immunized chickens to develop a certain degree of resistance to coccidial infection.

To investigate the types of the intestinal microbiota involved in resistance to *Eimeria*, the intestinal contents and fecal samples from the immunized and unimmunized groups were collected and subjected to 16S rRNA gene sequencing. Bioinformatics analyses were then performed to compare the differences between the gut microbial composition of the two groups.

Following the preliminary analysis of microbial richness and diversity indices, the microflora structure in the cecum content and feces was analyzed at the phylum level. According to previous studies, at the phylum level, the cecal environment mainly comprises Firmicutes, Bacteroidetes, Proteobacteria, and Desulfobacterota; this finding is consistent with previous reports [9], indicating that most of these groups belong to strictly anaerobic bacteria. As shown earlier, *Bacteroides* and Firmicutes play a crucial role in the metabolic process of various substances, and Firmicutes can maintain the stability of the immune system [28]. Firmicutes and *Bacteroidetes* can generate short-chain fatty acids (SCFAs), such as lactic acid and acetic acid, through fermentation [29,30]. They also regulate bile acid metabolism, collectively contributing to the equilibrium of the intestinal microbiota and playing crucial roles in host health and disease [30,31,32]. *Bacteroidetes* and Firmicutes are important microbiota in the intestine [33]. Firmicutes and *Bacteroidetes* have functions closely related to carbohydrate and protein metabolism, and they play a role in energy production [34,35]. Additionally, some members in the Firmicutes phylum can regulate inflammation by inducing anti-inflammatory cytokines [36]. As shown in previous studies, following *E. tenella* infection, the abundance of Firmicutes in the cecum significantly decreases, which may be one of the reasons for malnutrition and weight loss in chickens [37]. However, in the present study, after multiple rounds of low-dose immunization, the abundance of Firmicutes in the cecal content of the immunized group significantly increased (from 67.44% to 73.01%) and that of Bacteroidota decreased significantly (from 29.77% to 24.44%) as compared to those of the unimmunized group; this may contribute to the development of the ability of chicken cecum to resist coccidial infections. In particular, in the feces of immunized chickens, the Firmicutes proportion increased significantly (from 67.44% to 95.96%), the Proteobacteria proportion increased slightly (from 1.17% to 2.12%), and the *Bacteroidetes* proportion decreased significantly (from 29.77% to 1.47%). A high proportion of Firmicutes (95.96%) may be one of the key factors for the immunity afforded by transplantation of the fecal solution in the FMT experiment.

LEfSe analysis indicated that the abundance of *Eubacterium coprostanoligenes* (*E. copr*), *Erysipelatoclostridium*, *Shuttleworthia*, and *Colidextribacter* increased in the gut microbiota after immunization. *E. copr* is a core genus in the gut. Bai et al. showed that *E. copr* can alleviate chemotherapy-induced intestinal mucositis and further emphasized its role in regulating gut mucus secretion, which is critical for effective microbial defense [38]. Wei et al. observed that *E. copr* mediates lipid-lowering effects in high-fat diet (HFD) through sphingolipids, and supplementation of sphingolipids improved HFD-induced dyslipidemia [39]. Li et al. found that *E. copr* can colonize the chicken gut [40], and further research showed that the oral administration of *E. copr* in mice can reduce cholesterol levels [41]. In the present study, the abundance of Clostridiales and Clostridiaceae was increased in the fecal microbiota of the immunized group. *Clostridia*, belonging to the phylum Firmicutes, is one of the most abundant symbiotic bacterial species in the intestine. Most of its members are Gram-positive anaerobes and producers of SCFAs [42]. Members of Lachnospiraceae and *Ruminococcaceae*, both belonging to the order Clostridiales, can regulate acetylated H3 levels in CD4+ T cells and maintain the regulatory T cell (Treg)/Th17 ratio. Their metabolites (SCFAs) induce immune tolerance through Treg cells [43]. These findings suggest that *E. copr* and Clostridiales may be dominant bacterial strains conferring resistance to *E. tenella* infection.

## 5. Conclusions

The present study findings demonstrate that the gut and fecal microbiota of chickens with complete resistance to *E. tenella* infection undergo significant changes. Specifically, the abundance of *E. copr*, *Erysipelatoclostridium*, *Shuttleworthia*, and *Colidextribacter* increased in the gut microbiota after immunization, while the abundance of Clostridiales and Clostridiaceae was increased in the fecal microbiota. Chickens inoculated with fecal microbiota from immunized birds through FMT demonstrated partial resistance to *E. tenella*. These bacterial groups may represent key strains that contribute to resistance against *E. tenella* infection. Further research should focus on isolating these potential beneficial strains with anticoccidial effects and investigating the mechanisms underlying these host-parasite-microbiota interactions; this could help reveal key targets and novel therapeutic approaches for restoring gut microbiota and combating coccidiosis.

## Figures and Tables

**Table 1:** OPG values and cecal lesions scores after *E. tenella* challenge.

Groups	OPG			Cecal Lesions Scores
	6th d	7th d	Average	
Immunized challenge group	0	0	0 ^a^	0 ^a^
Unnimmunized challenge group	78,000 ± 5400	915,000 ± 72,000	496,500 ± 33,000 ^b^	2.0 ± 0.47 ^b^
Unnimmunized unchallenge group	0	0	0 ^a^	0 ^a^

^a, b^ Different letters in the same column indicate significant differences (*p* < 0.05). OPG: oocysts per gram feces.

**Table 2:** Protective effect of gut microbiota against *E. tenella* infection.

Groups	OPG				Oocyst Reduction Rate (%)	Cecal Lesions Scores
	6th d	7th d	8th d	Average		
Cloaca 1 g	1500 ± 400	405,000 ± 36,000	21,900 ± 2100	142,800 ± 21,000 ^bc^	8.6	2.9 ^d^
Cloaca 5 g	900 ± 70	216,000 ± 22,000	105,000 ± 1800	107,300 ± 18,000 ^d^	31.3	2.4 ^bc^
Cloaca 10 g	138,000 ± 16,200	216,000 ± 24,000	60,000 ± 4500	138,000 ± 21,000 ^c^	11.7	2.1 ^b^
Gavage 1 g	7800 ± 700	192,000 ± 18,000	61,500 ± 4900	87,100 ± 7200 ^e^	44.2	2.3 ^bc^
Gavage 5 g	16,100 ± 500	403,500 ± 29,000	19,000 ± 2300	146,200 ± 19,000 ^b^	6.4	2.5 ^bcd^
Gavage 10 g	18,000 ± 5600	394,800 ± 35,000	138,000 ± 1900	142,200 ± 22,000 ^bc^	9	2.7 ^cd^
unimmunized-challenge	600 ± 100	414,000 ± 39,000	54,000 ± 3700	156,200 ± 19,000 ^f^	0	2.9 ^d^
unimmunized-unchallenge	0	0	0	0 ^a^	-	0 ^a^

^a–f^ Different letters in the same column indicate significant differences (*p* < 0.05). Cloaca: Inoculation immunity via cloaca. Gavage: inoculation immunity via cloaca. OPG: oocysts per gram feces.

**Table 3:** Results of alpha diversity indices for the immunized and unimmunized groups.

Samples	Chao1	Goods_Coverage	Shannon	Observed_Species	Simpson	PD_Whole_Tree
CG_CC	2900.283	0.99	7.34	2427.2	0.98	83.406 ^a^
EG_IMC	2256.674	0.99	6.61	1802.2	0.95	67.329 ^b^
CG_CF	796.055	1	2.981	547.975	0.7	29.629
EG_IMF	884.336	1	2.999	680.966	0.68	36.409

^a, b^ Different letters indicate significant differences (*p* < 0.05).

## Data Availability

The raw sequencing data can be found at the National Centre for Biotechnology Information (NCBI) Sequence Read Archive (SRA) with an accession number: PRJNA1170940.
